# Nrf2 Regulates Oxidative Stress and Its Role in Cerebral Ischemic Stroke

**DOI:** 10.3390/antiox11122377

**Published:** 2022-11-30

**Authors:** Lei Wang, Xu Zhang, Xiaoxing Xiong, Hua Zhu, Ran Chen, Shudi Zhang, Gang Chen, Zhihong Jian

**Affiliations:** Department of Neurosurgery, Renmin Hospital of Wuhan University, Wuhan 430060, China

**Keywords:** Nrf2, cerebral ischemic stroke, oxidative stress, inflammation, mitochondrial function, blood–brain barrier, ferroptosis

## Abstract

Cerebral ischemic stroke is characterized by acute ischemia in a certain part of the brain, which leads to brain cells necrosis, apoptosis, ferroptosis, pyroptosis, etc. At present, there are limited effective clinical treatments for cerebral ischemic stroke, and the recovery of cerebral blood circulation will lead to cerebral ischemia-reperfusion injury (CIRI). Cerebral ischemic stroke involves many pathological processes such as oxidative stress, inflammation, and mitochondrial dysfunction. Nuclear factor erythroid 2-related factor 2 (Nrf2), as one of the most critical antioxidant transcription factors in cells, can coordinate various cytoprotective factors to inhibit oxidative stress. Targeting Nrf2 is considered as a potential strategy to prevent and treat cerebral ischemia injury. During cerebral ischemia, Nrf2 participates in signaling pathways such as Keap1, PI3K/AKT, MAPK, NF-κB, and HO-1, and then alleviates cerebral ischemia injury or CIRI by inhibiting oxidative stress, anti-inflammation, maintaining mitochondrial homeostasis, protecting the blood–brain barrier, and inhibiting ferroptosis. In this review, we have discussed the structure of Nrf2, the mechanisms of Nrf2 in cerebral ischemic stroke, the related research on the treatment of cerebral ischemia through the Nrf2 signaling pathway in recent years, and expounded the important role and future potential of the Nrf2 pathway in cerebral ischemic stroke.

## 1. Introduction

### 1.1. Cerebral Ischemic Stroke

Stroke refers to the obstruction or bleeding of arteries in a certain part of the brain tissue, which leads to focal neurological impairment in the corresponding part, including ischemic stroke and hemorrhagic stroke. Investigation showed that the incidence and prevalence of stroke increased with years, and stroke caused high rates of disability and mortality, which seriously endangered human life and health [1]. Among them, cerebral ischemic stroke makes up the vast majority, accounting for about 70% of all stroke events [2]. Cerebral ischemic stroke is featured with cerebral vascular congestion and insufficient blood supply, leading to insufficient supply of oxygen and nutrients in brain tissue, and then leads to a series of pathophysiological changes or injuries, and the death of brain cells, eventually leading to neurological dysfunction. At present, it is known that the pathophysiological changes caused by ischemic stroke include energy metabolism disorder of brain tissue, oxidative stress injury, excitatory amino acid poisoning, inflammatory reaction, etc. [3]. The ischemic parts of the brain are segmented into the ischemic core area and the penumbra area. The supply of glucose and oxygen in the ischemic core almost completely stops, resulting in irreversible damage to all cells and death due to necrosis. The penumbra can still acquire a small amount of glucose and oxygen supply from the blood flow, and prevent the cells from dying immediately [4]. If the blood flow can be restored in a timely manner, the function of brain cells may be restored. However, during the onset of acute stroke, partial opening of collateral blood circulation, or unobstructed blood circulation again after embolectomy, will cause ischemia-reperfusion injury, which will lead to the release of free radicals, which will lead to the destruction of brain cell structure and further aggravate brain tissue damage [5].

### 1.2. Nrf2

Transcription factor NF E2-related factor 2 (Nrf2) is a member of the transcription factor family, widely exists in various cells, and has redox sensitivity [6,7]. When cells are stimulated by oxidative stress, Nrf2 can regulate the antioxidant stress by combining with the antioxidant response element (ARE) in nucleus [8]. The cytoplasmic kelch-like epichlorohydrin-associated protein 1 (Keap1) can sense the oxidative insults, and Nrf2 will serve for the counter responses [9]. More than 600 genes, among them more than 200 encoding proteins linked to inflammation, cancer, neurological disorders, aging, cardiovascular disease, and other serious illnesses, are regulated by the Nrf2 signaling pathway [10]. Nrf2 can regulate most downstream factors, and it can play plenty of roles, including antiapoptosis, anti-inflammatory injury, reducing calcium overload, antioxidative stress, etc., and help the body maintain the redox reaction of brain tissue and brain cells. Nrf2 signaling is increasingly complex as Nrf2 is sited at the center of a vast regulatory network now. Thus, it is vital to stabilize Nrf2 activity to maintain the redox balance and brain homeostasis. As the brain is vulnerable to oxidative stress [11], Nrf2 activation has now become a promising target in the setting of cerebrovascular accidents such as ischemia. Nrf2 plays a crucial role in the management of excessive oxidative stress after stroke [12]. Hydrogen peroxide, which increases after ischemia-reperfusion, is the main stimulator of Nrf2 activation [13]. Nrf2^−/−^ rats have greater cerebral damage after ischemic stroke modeling due to the lack of Nrf2 protection [14]. The above results indicate that Nrf2 is an important molecule to regulate oxidative stress. Moreover, the expression level of Nrf2 can affect the recovery and prognosis of ischemic stroke injury.

### 1.3. Oxidative Stress

Free radicals of oxygen and nitrogen are necessary for all aerobic organisms. Reactive oxygen species (ROS) participate in the basic biochemical process of the organism, which can maintain the redox steady state of tissues and cells. The physiological health of the organism is very important. However, excessive oxidative stress may lead to lipid, protein, and DNA damage, which is harmful to the body. Oxidative stress means that the balance between the oxidative system and antioxidant system in the body is broken, which leads to the destruction of intracellular biological macromolecules such as sugars, lipids, protein, and nucleic acids, and further leads to the damage of organs, tissues, and functions, which in turn leads to the further accumulation of oxidation products, thus aggravating the damage and forming a vicious circle [15]. Oxidative stress includes exogenous and endogenous mechanisms. Endogenous stress usually comes from intracellular signaling pathways, metabolism, and inflammatory processes [16]. Different types of ROS will be produced by metabolism, including superoxide anion radical (O_2_^•−^), peroxy radical (ROO^•^), hydroxyl radical (HO^•^), as well as the non-free radical compounds such as hydrogen peroxide (H_2_O_2_) and singlet oxygen (^1^O_2_) [17]. At present, it is known that the action of free radicals is concentration-dependent; that is, ROS is beneficial to the body at normal physiological concentration, but harmful to the body when it exceeds a certain concentration [18]. If the content of ROS exceeds the cell tolerance level, it will oxidize DNA and induce DNA breakage and damage, which is common during the occurrence of tumors [19]. Oxidative stress participates in cerebral ischemia injury through many ways, which damages the normal function of cells, causes irreversible damage to cells, and causes cell necrosis and apoptosis [20]. After cerebral ischemia, oxidative DNA leads to cell damage, activates several death-related signal pathways, and then leads to programmed cell death, leading to neurological dysfunction [21]. The antioxidant system is the body’s response to all kinds of oxidative damage in order to reduce oxidative damage. After ischemic stroke, cells start the antioxidant defense function, and activate enzymes such as superoxide dismutase or heme oxygenase [22]. Superoxide dismutase (SOD) and glutathione (GSH) play an antioxidant role by maintaining the redox balance of tissues and cells [23]. However, brain tissue contains many unsaturated fatty acids and high concentrations of lipids, and its antioxidant capacity is weak, which makes brain tissue vulnerable to the damage of ROS [24]. Therefore, it is of great significance to study the mechanism of oxidative stress after cerebral ischemia.

### 1.4. The Role of Nrf2 in Cerebral Ischemic Stroke

Study has shown that cells significantly elevate the Nrf2 expression during the acute phase of stroke [25]. In the models of middle cerebral artery occlusion (MCAO), the expression of Nrf2 was upregulated from 3 h after occlusion, and reached its zenith at 24 h [26]. Other studies have shown that Nrf2 participates in the process of CIRI and has endogenous neuroprotective effects [27,28]. The activation of Nrf2 is caused by excessive ROS generation after cerebral ischemia. After Nrf2 activation, transcription of a series of antioxidant genes was initiated. Some studies confirmed that heme oxygenase-1 (HO-1) and NAD(P)H quinone oxidoreductase 1 (NQO1) expression increased after cerebral ischemia [29]. Nrf2 activator reversibly reduced the expression of SODs, and the overexpression of SOD1 or SOD2 significantly reduced the brain damage caused by cerebral ischemic stroke [30,31]. The mechanism underlying the protective effect of Nrf2 in cerebral ischemic stroke is not yet completely understood. It was shown that Nrf2 alleviates CIRI by activating the expression of antioxidant genes, defending against the damage caused by ROS, alleviating blood–brain barrier disruption and attenuating inflammation [32]. The study showed that following transient middle cerebral artery embolism in Nrf2 knockout mice, the basic and induced activation of antioxidant enzymes and detoxification enzymes were inhibited, the cerebral infarction area was larger, and the behavioral performance was worse than that in the control group [33]. All the above studies show that Nrf2 plays a vital role in alleviating ischemic stroke and CIRI, and most studies show that this role is a beneficial protective role.

## 2. The Structure and Function of Nrf2

**Figure 1:**
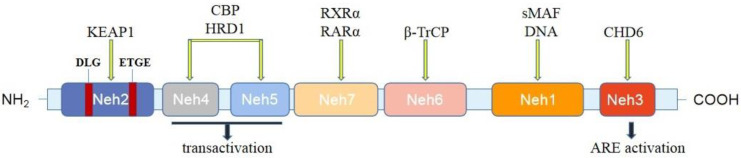
Domain structures of Nrf2. The Nrf2 protein contains seven domains: Neh1–Neh7. Neh2 is the Keap1-binding domain. Neh4, 5, and 3 domains are critical for transactivation. Neh3 can activate transcription of downstream-related genes. The Neh6 region participates in the Keap1 alternative degradation pathway. The Neh1 region binds to small musculoaponeurotic fibrosarcoma (sMaf) and forms a heterodimer. Neh7 can bind to RXRα and RaRα. KEAP1, cytoplasmic kelch-like epichlorohydrin-associated protein 1; CBP, cyclic AMP response element-binding protein; HRD1, HMG-CoA reductase degradation protein 1; RXRα, retinoid X receptor α; RARα, retinoic acid receptor α; β-TrCP, β-transducin repeat-containing protein; sMAF, small musculoaponeurotic fibrosarcoma; CHD6, chromodomain helicase DNA-binding protein 6; ARE, antioxidant response element.

The relative molecular weight of Nrf2 protein was 6.6 × 10^4^ Da. It is a transcription factor with a highly conserved basic leucine zipper structure, and is regulated by Keap1 at a low level and has a short half-life in non-activated cells [34]. Nrf2 mainly has seven highly conserved epichlorohydrin-related protein homologous domains (Nrf2 epichlorohydrin homology, Neh) [10,35] (Figure 1): The Neh1 region is a leucine zipper motif associated with intranuclear small musculoaponeurotic fibrosarcoma (sMaf), forms a heterodimer, recognizes and binds related sequences on the ARE, and can also interact with E2 ubiquitin-binding enzyme to adjust Nrf2 stability. The Neh2 region is a Keap1-dependent regulatory region, which contains two binding units, DLG and ETGE, to negatively regulate the Nrf2 transcriptional activity and promote its degradation [36]. The DLG-binding motif of Nrf2 functions as a “latch” separating from Keap1, while the ETGE-binding motif acts like a ‘hinge’ that remains connected to Keap1 [37]. Neh3 is located at the carboxyl terminus of Nrf2 and acts as a transactivation domain to activate transcription of related genes downstream [38]. Neh4 and Neh5 are two independent activation regions, both of which are rich in acidic amino acid residues and participate in the regulation of Nrf2 transcription by binding to cyclic AMP response element-binding protein (CREB) [39]. The Neh6 region is a Keap1 independent regulatory region and participates in the Keap1 alternative degradation pathway. Neh7 can specifically interact with retinoic acid X receptor α (RXRα) [40]. Brain microglia, macrophages, and astrocytes are the main producers of Nrf2; they produce a large amount of Nrf2 during the first 24 h after the induced middle cerebral artery occlusion [41,42].

Normally, Nrf2 forms a complex with Keap1, a negative regulator of Nrf2, at a low expression level. Nrf2 will disconnect from Keap1 under oxidative stress, and then Nrf2 will help to transfer Keap1 into the nucleus. Nrf2 possesses protective effects against oxidative stress and inflammation [43]. Nrf2 can adjust the expression of antioxidant enzymes, including catalase (CAT), glutathione peroxidase (GSH-Px), and SOD, and it can regulate the expression of some phase II detoxification enzymes, such asNQO1, HO-1, and glutathione S-transferase (GST) [44,45]. Many Nrf2-driven genes become involved in glutathione synthesis or action. The de novo synthesis of glutathione starts with γ-glutamylcysteine synthetase (γ-GCS), which binds cysteine with glutamate to produce γ-glutamylcysteine. HO-1 is one of the crucial and well-studied antioxidant genes adjusted by Nrf2 and is of particular importance in endothelial homeostasis. HO-1 is usually upregulated along with ferritin. HO-1 combines with biliverdin reductase to release bilirubin, which ranks as one of the most robust endogenous antioxidants by clearing reactive oxygen species and reactive nitrogen species ROS/RNS [46].

In addition, other downstream classes of genes involved in protein transport, ubiquitination, phosphorylation, proliferation, and apoptosis, have also been shown to be potentially Nrf2-regulating endogenous encoding genes [47]. Nrf2 has also been shown to regulate genes in response to air pollution or increased reactive oxygen species [48]. Nrf2-related signaling pathways play an important role in maintaining cellular homeostasis under stress, inflammation, carcinogenesis and proapoptotic conditions [49,50]. This shows that Nrf2 is widely involved in the physiological and pathological processes of the body, and it is an important cytokine.

In addition to regulating cellular oxidative stress response, Nrf2 is also involved in the regulatory process of maintaining intracellular redox homeostasis. By activating the expression of various kinds of antioxidant proteins, Nrf2 can reduce ROS-induced cellular damage and electrophilics, and maintain a redox dynamic balance in the human body. Under oxidative stress, the content of ROS in vivo increases and the Nrf2 system is activated, the cells express more antioxidant enzymes and proteases of synthetic antioxidants, the antioxidant mechanism is enhanced, and the content of ROS decreases, thus achieving the dynamic redox balance [51]. Therefore, Nrf2 is an important factor to maintain cell homeostasis and organism homeostasis.

## 3. Oxidative Stress and Cerebral Ischemic Stroke

Oxidative stress refers to the excessive production of ROS and reactive nitrogen species (RNS) when the body is under many external or internal stimuli, and the imbalance of oxidation system and antioxidant system leads to cell damage. The main mechanism of oxidative stress injury is the increase of free radical sources and the decrease of antioxidant capacity. ROS are the oxygenated molecules with high activity properties, including O_2_^•−^, H_2_O_2_, NO, and ONOO^−^, etc. In living cells such as neurons, ROS can be produced in response to hypoxia, serum deprivation, or cytokine stimulation [52]. Mitochondria are the main organelles that generate ROS in cells [53]. RNS refers to the redox molecule derived from nitric oxide (NO). NO is generated by NOS catalyzing the L-arginine guanidine-based terminal nitrogen oxidation reaction. Low concentrations of NO can resist oxidative damage, while high concentrations of NO can aggravate the oxidative damage of the body [54,55]. Oxidative stress is known to be involved in several pathophysiological processes [56]. Therefore, the regulation of oxidative stress is an important factor to maintain the balance of the body.

After CIRI, the blood–brain barrier of brain tissue is destroyed, and oxidative stress is one of the factors [57]. It has been found that oxidative stress is a significant mechanism leading to brain tissue injury after cerebral ischemic stroke [58,59]. In CIRI, when the generation of oxidants surpasses the body’s antioxidant capacity, the accumulated ROS will trigger the oxidation reaction of DNA, lipid, and protein, leading to the death of tissue cells, and then cause neurological dysfunction [60]. However, during reperfusion, a great deal of reactive oxygen species, such as hydroxyl radicals and superoxide radicals, and an excess increase in hydrogen peroxide, are produced [61]. ROS tend to affect cell membranes lipids, and lipid peroxidation will generate a vast number of lipid peroxides (4-HNE and other toxic aldehydes), which increase the cell membranes’ permeability and destroy their structural integrity [62]. ROS and RNS interact to produce reactive oxygen and nitrogen molecules, such as strong oxidant peroxynitrite. It has been found that NADPH oxidase 2 (NOX2) plays a key role in CI/R-induced oxidative stress injury and is a key enzyme causing ROS [63]. In addition, several studies showed that decreasing NOX2 expression and reducing NOX activity are two effective ways to attenuate CIRI [64]. When studying the protective mechanism of ischemic preconditioning, some researchers found that ischemic preconditioning (IPC) can induce cerebral ischemic tolerance, which is initiated by oxidative stress. This mechanism is related to the opening up of the adenosine triphosphate (ATP)-sensitive potassium channel (mito K^+^ ATP), which is necessary for IPC to play a protective role. Moreover, it was found that there is a delicate balance in ROS generation; for instance, a high level of ROS produced during IR is cytotoxic, while a low level of IPC-produced ROS is neuroprotective [65].

The SOD family includes SOD1, SOD2, and SOD3 proteins that neutralize the first oxygen-sourced ROS. SOD belongs to the body of free radical scavenging agents, and protect cells against oxidative stress, especially in the removal of superoxide produced during metabolism [66]. In addition, SODs can inhibit the damage of oxygen free radicals, and also reduce the number of peroxidation products generated by brain tissue under ischemia and reperfusion, and increase the ability of the cerebral cortex to withstand hypoxia [67]. This shows that SODs have a protective effect on ischemic brain injury. Moreover, the balance between SODs and ROS is very important for the homeostasis of the body. If the balance between them cannot be maintained, the cell homeostasis will be destroyed.

Mitochondria are the energy factories of cells. Excessive ROS has a highly toxic effect on key cellular macromolecules, and ROS can damage the mitochondria, leading to disorder of the cellular energy metabolism and ultimately cell death [68,69]. After stroke, the blood supply of local brain tissue is insufficient, mitochondrial dysfunction occurs, ATP cannot be generated, and calcium homeostasis is disrupted. After cerebral blood flow reperfusion, oxidative stress further aggravates mitochondrial damage [70]. This is followed by apoptosis and, in turn, mitochondrial dysfunction produces excess ROS [71]. The mechanism may be that under the condition of cell hypoxia, the function of mitochondria to produce ATP is inhibited, thus affecting the normal function of the Na(+)/K(+)-ATPase and Ca^2+^/H^+^-ATPase plasma pump, the increase of intracellular Na^+^, Ca^2+^, and adenosine diphosphate (ADP) content, the disruption of cell ion homeostasis, and the depolarization of the cell membrane, leading to the change of intracellular mitochondrial membrane potential and the release of a large amount of ROS. SOD and GSH-Px are endogenous antioxidant enzymes that can clear free radicals and protect cells from oxidative injury. After cerebral ischemic stroke, the activities of SOD and GSH-Px were significantly decreased, resulting in serious damage to the structure and function of mitochondria, and inducing brain tissue damage and neurologic function deficits [72]. The above results demonstrate that ROS and mitochondrial damage interact and promote each other during stroke.

More and more researches have shown that oxidative stress is involved in stroke and CIRI; therefore, many drugs take effect by regulating oxidative stress, especially some traditional Chinese medicines or compounds. This paper summarizes that these drugs play a protective role in stroke and CIRI by regulating oxidative stress in the past five years. Dihydrocapsaicin (DHC) is the main active substance of capsaicin in chili peppers. The study showed that DHC can protect the brain and blood–brain barrier from I/R damage by inhibiting oxidative stress and inflammation [73]. Acteoside (ACT) has neuroprotective and antioxidant effects in neurodegenerative diseases. The results showed that ACT could reduce oxidative stress in the middle cerebral artery occlusion/reperfusion (MCAO/R) models [74]. Schizandrin A (Sch A) is isolated from Schisandra chinesnesis and it has significant antioxidant activities. It has been shown that Sch A has a protective role against cerebral stroke [75]. One of the major active ingredients of Herba Epimedii, Icariside II, can attenuate CIRI through inhibiting oxidative stress [76]. Rutaecarpine (Rut) is an alkaloid isolated from Evodia officinalis and has many biological activities. Rut can inhibit inflammation, oxidative stress, and apoptosis, and it can attenuate CIRI [77]. Biochanin A is a natural phytoestrogen. The results showed that biochanin A had protective effects on cerebral ischemic injury by antioxidation in rats [78]. Studies have proven that Theaflavin possesses strong antioxidative capacity. Theaflavin can reduce oxidative stress, thereby attenuating cerebral ischemia-reperfusion injury [79]. Carvacryl acetate (CA) is a semi-synthetic monoterpenic ester extracted from essential oils, which has antioxidant properties. The study has shown that CA can alleviate oxidative stress injury induced by cerebral ischemia-reperfusion through the Nrf2 signaling pathway [80]. Convolvulus pluricaulis Choisy is traditionally prescribed for nerve debility. The study showed that Convolvulus pluricaulis Choisy has a neuroprotective effect on the oxidative stress model of CIRI in rats [81]. Cepharanthine (CEP) has anti-inflammatory and antioxidative properties. It has been shown that CEP attenuates cerebral I/R injury by inhibiting nod-like receptor family pyrin domain-containing 3 (NLRP3) inflammasome-induced inflammation [82]. Studies have shown that Sanggenon C (SC) has antioxidant effect. The results showed that SC can inhibit inflammation and oxidative stress in CIRI [83]. Coicis Semen has antioxidative roles. Coicis Semen can alleviate ischemic brain injury, which may be related to the inhibition of oxidative stress [84]. It has been shown that resveratrol has an anti-inflammatory effect. Pic can reduce oxidative stress and apoptosis caused by CIRI, and the effect is realized by adjusting the Sirtuin1 (SIRT1)/fork-head box O1(FoxO1) signaling pathway [85]. Garcinol is a polyisoprenylated benzophenone derivative. Study has shown that Garcinol can inhibit oxidative stress and improve cerebral ischemia injury [86]. Fisetin has an antioxidant and anti-inflammatory effect. Fisetin protected CIRI injury, perhaps due to suppression of oxidative stress and inflammatory reaction [87]. Geraniin is a polyphenol isolated from Phyllanthus amarus. Study has shown that Geraniol can protect against CIRI by inhibiting oxidative stress [88]. Cucurbitacin B (CuB) has been demonstrated to possess antioxidative properties. The study showed that CuB can decrease lactate dehydrogenase (LDH) release and ROS production; that is, it can reduce cerebral I/R injury by reducing the level of oxidative stress [89]. Fraxin, one of the primary active ingredients of Cortex Fraxini, may have potent anti-inflammatory activity. A recent study showed that Fraxin significantly improved CIRI by inhibiting oxidative stress and inflammatory response [90]. Scutellarin can protect against cerebral ischemia injury, and study has shown that this is related to its antioxidant effect [91]. These drugs play a protective role in cerebral ischemic stroke or CIRI by regulating oxidative stress, and their specific regulatory mechanisms are different. We summarized these studies, and showed them in the table (Table 1) below.

## 4. Activation of Nrf2 Signaling Pathway in Cerebral Ischemic Stroke

### 4.1. Keap1/Nrf2/ARE Signaling Pathway

Keap1 (kelch-like ECH-associated protein 1) is an important regulatory protein. It forms a complex with ubiquitin protein kinase Cul3/rbx1 through specific binding with the target protein, and mediates the ubiquitination and degradation of the target protein. The primary structure of Keap1 contains 624 amino acids, including five domains: DGR, NTR, BTB/POZ, IVR, and CTR [92]. Among them, the binding site of Keap1 and Neh2 is located in the DGR region of the C-terminus, which is a repetitive fragment with six double-chain glycines. It is an Nrf2 inhibitory polypeptide and plays a role in inhibiting the translocation of Nrf2 into the nucleus as a negative regulatory protein. BTB/POZ is involved in the dimerization process of Keap1 protein and can enhance the binding force of Nrf2-Keap1. The core site of the BTB/POZ region is Ser-104, and its mutation will affect the dimerization of Keap1 and, thus, interfere with the ability of Keap1 to bind Nrf2 [93]. IVR contains 25 cysteine residues and is a region that regulates Keap1 activity. It can trigger intracellular redox reaction and contribute to the maintenance of intracellular redox balance [94].

**Figure 2:**
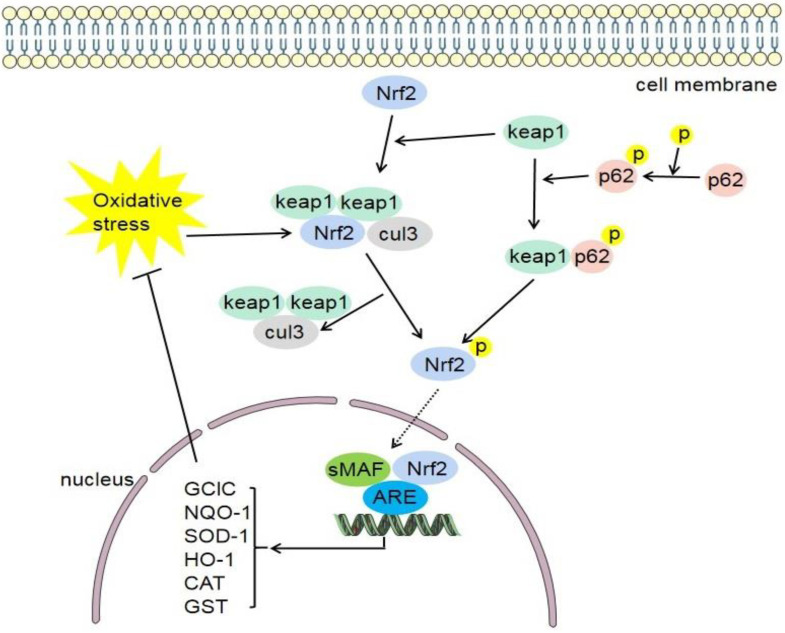
Keap1-Nrf2-ARE signaling pathway. Under normal physiological conditions, Keap1 binds to Nrf2 and makes it inactive. When oxidative stress occurs in the body, Nrf2 and Keap1 are dissociated and activated, transferred to the nucleus, and combined with the ARE in the nucleus. Furthermore, they can promote the activation of various target genes (GCLC, NQO-1, SOD-1, HO-1, CAT, GST, etc.) and inhibit oxidative stress in feedback. Keap1, cytoplasmic kelch-like epichlorohydrin-associated protein 1; Nrf2, nuclear factor erythroid 2-related factor 2; sMAF, small musculoaponeurotic fibrosarcoma; ARE, antioxidant response element; cul3, cullin-3; GCLC, glutamate–cysteine ligase catalytic; NQO1, NADPH quinone oxidoreductase enzyme; SOD-1, superoxide dismutase-1; HO-1, heme oxygenase-1; CAT, catalase; GST, glutathione S-transferase.

Under physiological condition, Nrf2 and Keap1 exist in the cytoplasm, and the inactive Nrf2 is ubiquitinated and then degraded. When oxidative stress occurs in the body, Nrf2 and Keap1 are dissociated and activated, transferred to the nucleus, and combined with the corresponding sites of the ARE in the nucleus, thereby activating the transcription of antioxidant enzymes and detoxification enzymes downstream of the pathway (Figure 2), and working with related factors to improve the antioxidant capacity of cells [95]. Keap1 contains an NES in the IVR domain. Oxidative stress can inhibit NES activity [96]. DJ-1 promotes Nrf2 transport to the nucleus by inhibiting Nrf2 ubiquitination and preventing its binding to Keap1 [97]. Nrf2 mediates the transcriptional activation of antioxidant enzyme genes such as HO-1, NQO1, and those belonging to the GST family [98]. Studies have shown that the Keap1-Nrf2 signaling pathway is one of the main defense mechanisms against oxidative stress through the regulation of cell protective gene expression [7]. The following research shows that some Nrf2 activators will not affect the binding of Keap1 and Nrf2 [37]. In parallel with the experimental approaches focusing on the oxidative stress-induced regulation of Nrf2, a cDNA expression screen for activators of Nrf2-dependent gene expression identified the autophagy adaptor protein p62 as a novel regulator of ARE gene expression [99]. P62 plays a role in many cellular functions, and p62 can activate Nrf2. The phosphorylation of p62 can increase the binding affinity of Keap1 [100]. Recent study has shown that p62 can not only competitively bind to Keap1, but also directly promote the degradation of Keap1 through selective autophagy [101].

The Keap1/Nrf2 signaling pathway is the most important antioxidant defense pathway found at present, which can resist cellular oxidative stress injury and play an important role in neurological diseases [102]. Trilobatin (TLB) is a naturally occurring SIRT3 agonist, and TLB can regulate neuroinflammation and oxidative reaction by regulating the TLR4/nuclear factor-kappa B and Nrf2/Keap1 signal pathway, reducing neuroinflammation and oxidative damage induced by CIRI, and playing a neuroprotective role [103]. In a cerebral ischemia model, the researchers observed the downregulation of MicroRNA-139-5p (miR-139-5p) and the pyroptosis induced by the activation of NLRP3. In addition, it was found that Ginsenoside Rd (Rd) played a protective role by regulating the ROS/TXNIP/NLRP3 inflammasome axis. These findings indicate that Rd can protect against ischemic stroke by regulating the FoxO1/Keap1/Nrf2 pathway [104]. Polyphenol-rich fraction (PRF) has an obvious protective effect on cerebral ischemia injury, which is manifested in PRF’s ability to improve the neurological deficit after transient middle cerebral artery occlusion (tMCAO) and to reduce the infarction rate and improve the cell morphology of the hippocampal CA1 area. PRF regulated oxidative stress by regulating the Keap1/Nrf2/HO-1 pathway and exhibited a good protective effect against CIRI [105]. To summarize, Keap1/Nrf2 signaling pathway is an effective mechanism to regulate brain injury.

### 4.2. Phosphatidylinositol-4,5-Bisphosphate 3-Kinase (PI3K)/Protein Kinase B (Akt)-Nrf2 Signaling Pathway

The PI3Ks are a family of lipid kinases. The PI3K/Akt pathway is associated with proliferation, cancer, and longevity. Akt is a serine/threonine kinase, which serves an important role in apoptosis, cell proliferation, transcription, and cell migration. There are three Akt subtypes: PKBα (Akt1), PKBβ (Akt2), and PKBγ (Akt3) [106]. In general, PI3K is mainly activated directly and indirectly through the focal adhesion kinase (FAK) pathway. Akt is the downstream signal molecule of PI3K [107].

**Figure 3:**
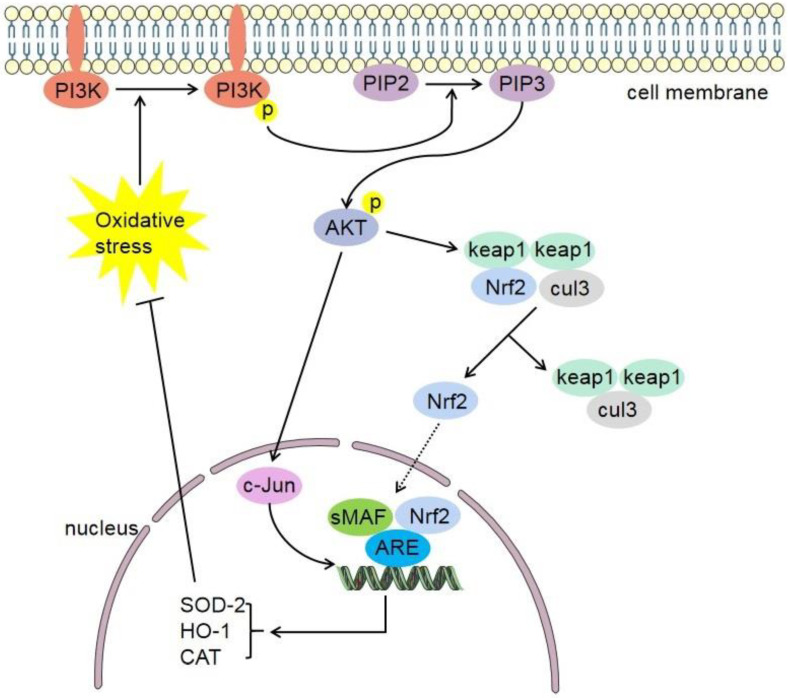
PI3K/Akt-Nrf2 signaling pathway. Under oxidative stress conditions, the phosphorylation expression of PI3K increases. Akt is a downstream target of PI3K. Akt promotes the dissociation of Nrf2 and Keap1 and transfers them to the nucleus. Nrf2 translocates into the nucleus, where in it heterodimerizes with small Maf proteins (sMaf) and binds to an enhancer sequence termed ARE. Furthermore, Nrf2 can promote the activation of various target molecules (HO-1, CAT, SOD-2, etc.) and inhibit oxidative stress in feedback. AKT can also promote the expression of C-Jun and the combination of Nrf2 with sMaf and ARE. Keap1, cytoplasmic kelch-like epichlorohydrin-associated protein 1; Nrf2, nuclear factor erythroid 2-related factor 2; sMAF, small musculoaponeurotic fibrosarcoma; ARE, antioxidant response element; PI3K, phosphatidylinositol 3-kinase; PIP2, Lipid phosphatidylinositol 4,5-bisphophate; PIP3, Phosphatidylinositol 3,4,5-triphosphate; cul3, cullin-3; HO-1, heme oxygenase-1; SOD-2, superoxide dismutase-2; CAT, catalase.

Studies have shown that the PI3K/Akt pathway plays a key role in ischemic damage of other organs. Studies have shown that hyperbaric oxygen preconditioning protects myocardial ischemia-reperfusion injury, and its effect is related to the regulating of the PI3K/Akt/Nrf2 pathway [108]. PI3K/Akt signaling pathway is involved in Nrf2 translocation, and upregulation of PI3K and Akt can activate Nrf2 [109,110]. Studies on ischemic stroke have found that the PI3K/Akt signaling pathway can promote cell survival and inhibit cell apoptosis, and play an important role in neuroprotection during cerebral ischemia-reperfusion [111]. Compared with a model group, the cognitive impairment and neurological deficits of a Rehmannioside A group were significantly improved, and the protective role was related to the inhibition of ferroptosis and activation of the PI3K/AKT/Nrf2 pathway [112]. Another study showed that diterpene ginkgolides meglumine injection (DGMI) could improve CIRI by activating Nrf2 mediated by PI3K/Akt [113]. A study showed that 6′-*O*-galloylpaeoniflorin (GPF) could improve the neurological deficit of CIRI rats. Further research shows that this process can be inhibited by PI3K inhibitor Ly294002. In conclusion, GPF possesses neuroprotective effects against oxidative stress after CIRI by activation of the PI3K/Akt/Nrf2 pathway [114]. A study showed that hydrogen sulfide (H2S) preconditioning could protect mice against CIRI by regulating the PI3K/Akt/Nrf2 pathway [115]. The role of the PI3K/Akt-Nrf2 signaling pathway in cerebral ischemia stroke is shown in Figure 3.

### 4.3. MAPK/Nrf2 Signaling Pathway

**Figure 4:**
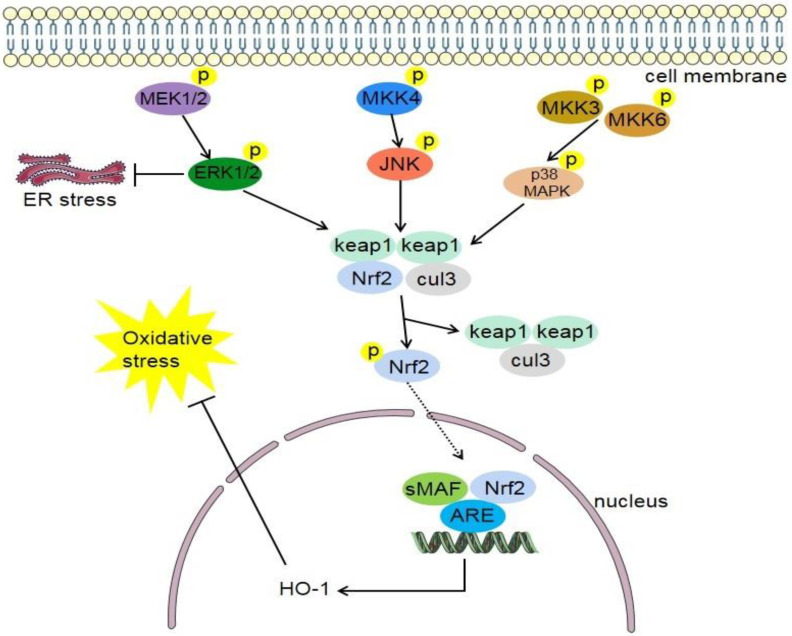
Mitogen-activated protein kinase (MAPK) pathway. It consists of three pathways which involve extracellular signal-regulated kinase 1 and 2 (ERK1/2), c-Jun N-terminal kinase (JNK), and p38 MAPK signaling pathways. Phosphorylated MEK1 and MEK2 promote the expression of ERK1 and ERK2, and then promote the dissociation of Nrf2 and Keap1. Phosphorylated MKK4 promotes the expression of JNK, and then promotes the dissociation of Nrf2 and Keap1. Phosphorylated MKK3 and MKK6 promote the expression of p38 MAPK, and then promote the dissociation of Nrf2 and Keap1. Nrf2 translocates into the nucleus, wherein it heterodimerizes with small Maf proteins (sMaf) and binds to an enhancer sequence termed ARE. This, in turn, promotes the expression of HO-1 and Oxygenase-1, and inhibits oxidative stress in feedback. MEK, mitogen-activated protein kinase; MKK4, MAPK kinase 4; MKK3, MAPK kinase 3; MKK6, MAPK kinase 6; ERK1/2, extracellular signal-regulated kinase 1 and 2; JNK, c-Jun N-terminal kinase; ER stress, endoplasmic reticulum stress; Keap1, cytoplasmic kelch-like epichlorohydrin-associated protein 1; Nrf2, nuclear factor erythroid 2-related factor 2; sMAF, small musculoaponeurotic fibrosarcoma; ARE, antioxidant response element; cul3, cullin-3; HO-1, heme oxygenase-1.

Mitogen-activated protein kinase (MAPK) can transmit extracellular signals from cell surface to nucleus. It is widely involved in a variety of signal transmission processes in the cell and plays a role in the activation and expression regulation of the Nrf2/HO-1 pathway [116]. MAPKs respond to a variety of stimuli, including a variety of endogenous and exogenous stress signals. Thus, they are traditionally classified in mitogen and stress activated MAPKs, with classic representatives being extracellular signal-regulated kinase (ERK) as mitogen responsive and C-Jun N-terminal kinase (JNK) and p38 as stress responsive MAPKs (Figure 4). p phosphorylates Nrf2 to separate it from Keap1 in the cytoplasm, thereby promoting Nrf2 activation [117]. p38-specific inhibitors prevent degradation of Keap1 and nuclear translocation of Nrf2 and subsequent expression of HO-1 by inhibiting phosphorylation of p38 [118].

Phosphorylated ERK is the activated form of ERK. Studies have shown that downregulating the expression levels of phosphorylated ERK and Nrf2 can prevent diseases related to oxidative stress and inflammatory response [119]. ERK 1/2 inhibitor can significantly inhibit quercetin-induced expression of Nrf2 and HO-1, indicating that ERK 1/2 activation is necessary for Nrf2 stabilization and HO-1 transcription [120]. The JNK family is encoded by three genes: JNK1, JNK2, and JNK3 [121]. Under the stimulation of oxidative stress or other injury effects, the body produces cytokines such as IL, which can activate MAPK molecules [122], promote the transfer of Nrf2 [123], and thus upregulate the expression of HO-1 [124]. Carbon monoxide (CO), the product of HO-1 degradation of heme, may regulate many MAPKs [125]. Through this series of reactions, the balance of oxidation and antioxidation, proinflammatory, and anti-inflammatory reactions is finally achieved in the body.

Studies have shown that some Chinese herbs or compounds play a protective role in ischemic lesions by regulating the MAPK/Nrf2 signaling pathway. Shengmai formula (SMF) is a traditional Chinese medicine with antioxidant properties. Pretreatment of a myocardial ischemia model with SMF significantly relieved heart injury, which was related to the regulation of the PI3K/AKT/p38 MAPK/Nrf2 signal pathway [126]. Studies have shown that during ischemic stroke, p38 MAPK is transferred from cytoplasm to nucleus, thus promoting apoptosis [127]. Another study showed that artesunate has a protective effect on cerebral ischemia injury, which may be regulated by activating the Nrf2-dependent p38 MAPK signaling pathway. Study showed that artesunate could inhibit the occurrence of CIRI by inhibiting oxidative and inflammatory processes, and the protective effect of artesunate is related to the activation of the p38 MAPK pathway [128]. The study showed that in the MCAO model, lupeol activated Nrf2, inhibited caspase-3 activity, inhibited phosphorylation of p38 MAPK, and played a protective role against cerebral ischemia [129].

### 4.4. Nrf2/Nuclear Transcription Factor-Kappa B (NF-κB) Signaling Pathway

NF-κB is widely involved in cell proliferation and differentiation [130]. It is a very classical signaling pathway in vivo and plays an important role in the processes of inflammation [131,132], cell proliferation [130], and oxidative stress [133]. The NF-κB family is mainly composed of five members: p65 (Rel A), Rel B, C-Rel, p52/P100, and p50/P105. Under normal conditions, the NF-κB family is usually associated with inhibitor κB(IκB) in the form of homo- or heterodimer binding. Members of the family then form stable NF-κB/IκB complex and exist in the cytoplasm in an inactive form. The main role of IκB protein is to shield the nuclear localization site of NF-κB dimer, and prevent it from entering the nucleus. On the other hand, the IκB in the nucleus can also dissociate NF-κB from the DNA binding site, bringing it back to the cytoplasm [134].

The transcription factor NF-κB is a key regulator involved in inflammation [132]. NF-κB participates in the pathophysiological process of ischemic stroke [135]. Protein arginine methyltransferase 5 (PRMT5) is a type of methyltransferase enzyme. In cerebral ischaemia/reperfusion (I/R) injury, PRMT5 activates the NF-κB/NLRP3 axis to play a pro-inflammatory and pro-pyroptotic role. The administration of the PRMT5 inhibitor LLY-283 alleviated the neurological deficit [136]. Fingolimod (FTY720) FTY720 has protective effect on rats with ischemic stroke injury, which is related to the inhibition of the p38 MAPK/NF-κB signal pathway [137]. The study showed that Luteoloside reduced neurological deficit and brain edema in MCAO rats. Its protective effect is related to the inhibition of the PPARγ/Nrf2/NF-κB pathway [138]. In the model of CIRI, Eriocitrin attenuated oxidative stress and inflammatory response in CIRI rats by regulating the Nrf2/HO-1/NF-κB pathway [139]. Another study showed that the protective effect of Dl-3-n-butylphthalide (NBP) on neuroinflammation in mice with CIRI was related to Nrf2. Nrf2 regulates the TLR4/MyD88/NF-κB pathway to participate in this neuroprotective effect [140].

**Figure 5:**
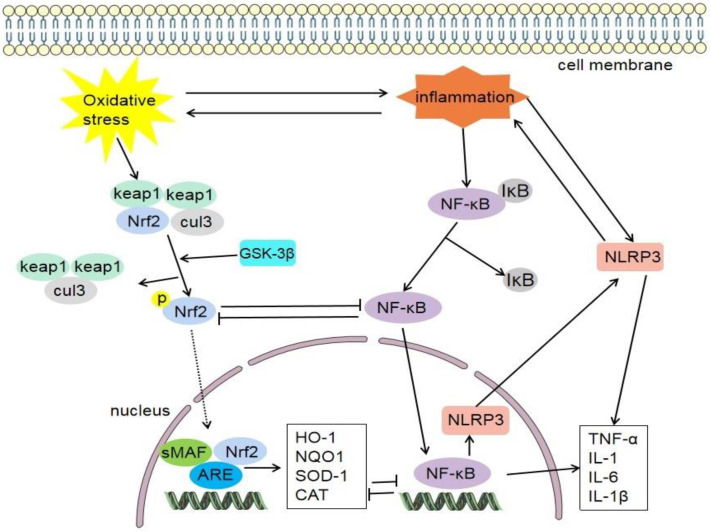
NF-κB is usually associated with IκB and forms stable NF-κB/IκB complex. Inflammatory reaction promotes the dissociation of NF-κB/IκB complex and separates active NF-κB. NF-κB can promote the expression of NLRP3, and then promote the inflammatory response. NF-κB can also promote the release of inflammatory factors (TNF-α, IL-1, IL-6, and IL-1β). Nrf2 can negatively regulate the NF-κB signaling pathway. Keap1, cytoplasmic kelch-like epichlorohydrin-associated protein 1; Nrf2, nuclear factor erythroid 2-related factor 2; sMAF, small musculoaponeurotic fibrosarcoma; ARE, antioxidant response element; cul3, cullin-3; NLRP3, nod-like receptor family pyrin domain-containing 3; HO-1, heme oxygenase-1; NQO1, quinine oxidoreductase 1; SOD-1, superoxide dismutase-1; CAT, catalase; NF-κB, Nuclear factor-kappa B; IκB, inhibitor κB; IL-1β, interleukin-1 β; TNFα, tumour necrosis factor-alpha.

Nrf2 can negatively regulate the NF-κB signaling pathway (Figure 5). The expression of toll-like receptor 4 (TLR-4) and NF-κB in Nrf2 knockout mice was higher than that of wild-type mice treated with tMCAO, and there were more severe neurological defects, infarct size, and inflammatory damage. Deletion of Nrf2 may lead to enhanced NF-κB activity and thus promote cytokine production, while NF-κB can also regulate the expression of Nrf2 in turn, and they crosstalk with each other. Keap1 plays a key role in the two signaling pathways [141].

### 4.5. Nrf2/HO-1 Signaling Pathway

**Figure 6:**
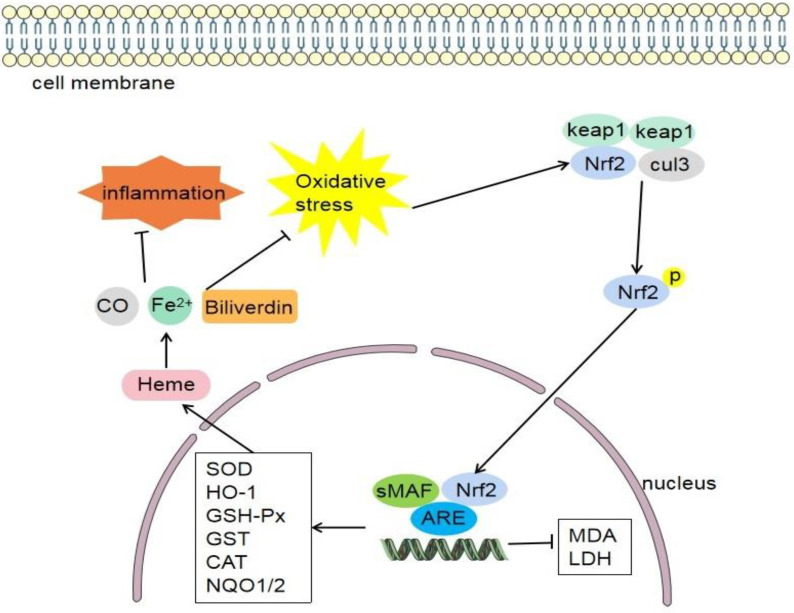
When oxidative stress occurs in the body, Nrf2 and Keap1 are dissociated and activated, transferred to the nucleus, Thereby promoting the activation of various target molecules (HO-1, SOD-1, GSH-Px, GST, CAT, NQO-1, NQO-2, etc.). Then, heme is further degraded into carbon monoxide (CO), biliverdin and iron ions (Fe^2+^), and the oxidative stress and inflammatory reaction are feedback inhibited. Keap1, cytoplasmic kelch-like epichlorohydrin-associated protein 1; Nrf2, nuclear factor erythroid 2-related factor 2; sMAF, small musculoaponeurotic fibrosarcoma; ARE, antioxidant response element; cul3, cullin-3; HO-1, heme-oxygenase-1; NQO, quinine oxidoreductase; SOD, superoxide dismutase; GSH-Px, glutathione peroxidase; GST, glutathione S-transferase; CAT, catalase; MDA, malondialdehyde; LDH, lactate dehydrogenase.

HO-1 is the rate limiting enzyme in the process of heme catabolism. It can degrade heme into CO, biliverdin, and iron ion (Fe^2+^) (Figure 6). These enzymatic products generally have anti-inflammatory and antioxidant effects [142], which are important mediators for Nrf2 to exert anti-inflammatory and antioxidant effects [143]. The Nrf2/HO-1 signaling pathway is an important mechanism for the body to defend against oxidative stress. Nrf2 can help maintain the physiological function of mitochondria, cellular redox reaction, and the normal function of proteins [144]. The expression of HO-1 gene is regulated by Nrf2. When Nrf2 is activated, it can promote the expression of HO-1. The upregulation of HO-1 expression can regulate these enzymes, such as SOD, GSH-Px, and CAT. Antioxidant enzymes can decompose free radicals in the body into water and molecular oxygen, reduce oxidative stress damage, and reduce the production of oxidation products, thus playing an anti-inflammatory and antioxidant role [143].

In recent years, more and more studies demonstrate that the Nrf2/HO-1 pathway can enhance the tolerance of brain tissue to ischemic oxidative damage [145,146]. As a target gene of Nrf2, HO-1 has a protective effect on brain injury. Studies have shown that pelargonidin can reduce cerebral ischemic volume, and improve memory and learning ability in MCAO rats. And the protective effect of pelargonidin is related to the activation of Nrf2/HO-1 signaling pathway [147]. Studies have shown that Rutaecarpine (Rut) can inhibit apoptosis, inflammation and oxidative stress, and then reduce CI/R-induced neuronal damage. And the protective effect is achieved by activating the expression of Nrf2/HO-1 pathway [77]. The activation of Nrf2/HO-1 signaling pathway can inhibit the expression of reactive oxygen species and pro-inflammatory factors, and significantly reduce the oxidative damage of ischemic brain tissue [148,149]. Wang. et al. demonstrated that when HO-1 decomposes heme, the produced CO can treat CIRI and permanent ischemic stroke stroke [150]. Geraniin is a kind of polyphenol isolated from Phyllanthus amarus. A study has shown that Geraniin can activate Nrf2/HO-1 signaling pathway, inhibit oxidative stress and neuronal apoptosis, and thus play a protective role in cerebral ischemia injury [88]. β-Caryophyllene (BCP) is a natural bicyclic sesquiterpene. Study has proved that BCP can improve the neurological function score, infarct volume after CIRI. The mechanism is related to the activation of Nrf2/HO-1 axis [151].

## 5. The Role and Mechanism of Nrf2 in Cerebral Ischemic Stroke

**Figure 7:**
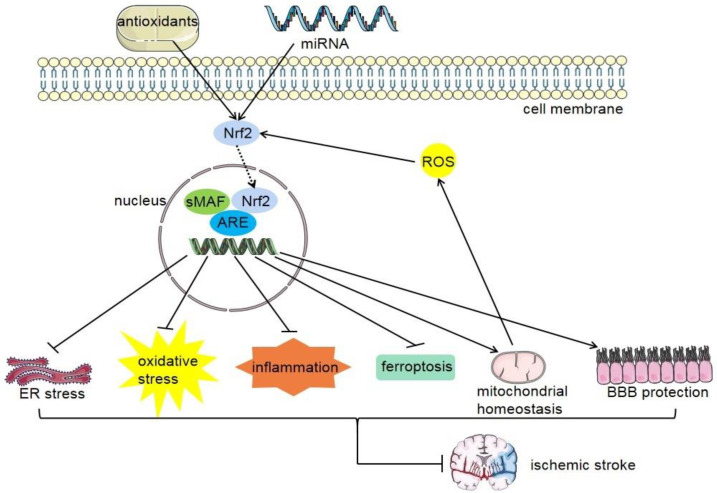
The beneficial impacts of Nrf2 activation on cerebral ischemia stroke. Antioxidants and miRNA can upregulate the expression of Nrf2 and then promote the expression of related target genes, which can inhibit ER stress, oxidative stress, inflammation, iron death, BBB, mitochondrial homeostasis, and brain protection.

Many studies have shown that Nrf2 is significantly overexpressed in the acute phase of stroke, and the content of Nrf2 is higher in the peri-infarct area than in the central area [152]. Compared with wild-type mice, after transient middle cerebral artery occlusion was established in Nrf2 knockout mice, the basic and induced activities of antioxidant enzymes and detoxification enzymes were decreased, the infarct area was larger, and the neurological function score was lower [153]. The mechanism of action of Nrf2 in cerebral ischemic stroke (Figure 7) may include the following points:

Nrf2, nuclear factor erythroid 2-related factor 2; sMAF, small musculoaponeurotic fibrosarcoma; ARE, antioxidant response element; ROS, reactive oxygen species; ER stress, endoplasmic reticulum stress; BBB, blood–brain barrier.

### 5.1. Nrf2 Regulates Oxidative Stress and Antioxidant Effect

Previous studies have confirmed that oxidative stress is a key mechanism for brain tissue injury during cerebral ischemic stroke [58,59]. Oxidative stress is induced by elevated production of ROS and RNS, which cause damage to all components of the cell, including proteins, lipids, and DNA [154]. The Nrf2 pathway is one of the important mechanisms involved in antioxidant stress. When oxidative stress occurs in cells, the Nrf2 signaling pathway is activated first, and then a large number of antioxidants and related enzymes are induced to reduce ROS production and resist cell damage caused by oxidative stress [155]. It has been confirmed that Nrf2 expression increases after cerebral ischemia and reperfusion (I/R), which induces the production of many endogenous antioxidant enzymes, such as NQO1, HO-1, SOD, GST, GSH-Px, etc., thus reducing or eliminating oxygen free radicals and improving the antioxidant capacity of cells and tissues [156].

Thioredoxin-1 (Trx1) is a redox regulatory protein that widely exists in organisms. The expression of Trx1 decreased during ischemic stroke. Adiponectin peptide (APNp) increased the expression of Trx1 and suppressed the activation of the NLRP3 inflammasome. Study has shown that APNp can reduce the volume of cerebral infarction, improve neurological function, and have antioxidant and antiapoptosis effects in a CIRI model [157]. Previous studies have shown that in the CIRI model, the transcription of Nrf2 and its downstream gene NQO1 in the treatment group increased, the activities of SOD and cat increased, and CIRI decreased. However, these protective effects were inhibited after knockdown of Nrf2. It indicates that the activation of Nrf2 can upregulate the activities of SOD, cat, and NQO1, thereby inhibiting oxidative stress and alleviating CIRI [75].

A study showed that after CIRI, the activities of SOD, GSH, and GSH-Px were significantly decreased and the level of MDA was significantly increased. However, phloretin pretreatment significantly inhibited these oxidative stress processes, reduced infarct volume, and improved neurological score. This indicates that phloretin exhibits neuroprotective effects in CIRI, and its mechanism is related to the inhibition of oxidative stress and the activation of the Nrf2 defense pathway [158]. Carvacryl acetate (CA) has an antioxidant effect. Studies have shown that CA can reduce CIRI in MCAO rats. The mechanism may be to increase the expression of Nrf2 and decrease the expression of ROS and MDA [80]. Biochanin A is a natural phytoestrogen. Biochanin A can enhance the activities of SOD and GSH-Px and inhibit the production of MDA. Biochanin A promotes nuclear translocation of Nrf2, promotes the expression of HO-1, and inhibits the activation of NF-kappaB. Pretreatment with Biochanin A can significantly alleviate brain injury and reduce infarct size and cerebral edema. Biochanin A protects the brain against ischemic injury through antioxidant and anti-inflammatory effects [78]. Geraniin may play a protective role against brain ischemia-reperfusion injury by regulating the Nrf2/HO-1 pathway and inhibiting oxidative stress and neuronal apoptosis [88]. Studies have shown that in cell models, diosmetin increased cell viability, decreased lactate dehydrogenase (LDH) release and ROS levels, and inhibited oxidative stress. In addition, diosmetin increased the protein expression of Nrf2, NQO1, and HO-1. This indicates that diosmetin can inhibit oxidative stress and alleviate CIRI through the SIRT1/Nrf2 signaling pathway [159].

### 5.2. Nrf2 Regulates Inflammation and Anti-Inflammatory Effects

Inflammation is a host defense mechanism triggered by injury and plays an important role in ischemic stroke [160]. In addition, the inflammatory process is mainly aseptic inflammatory reaction. Thus, endogenous damage-associated molecular patterns (DAMPs) are the exclusive trigger activating the innate immune system in ischemia [161]. The local excessive inflammatory reaction is accompanied by the process of cerebral ischemia, and it is also one of the main causes of brain tissue damage caused by cerebral ischemia [162]. A variety of inflammatory cells and inflammatory factors are involved in it. The main inflammatory cells involved are leukocytes, astrocytes, and microglia [161]. After cerebral ischemia, the blood–brain barrier (BBB) is destroyed, resulting in a large number of neutrophils infiltrating and attacking the brain [163,164]. In the acute phase of cerebral ischemia, inflammatory cells are infiltrated to produce different kinds of inflammatory factors, and participate in the main inflammatory mediators including cytokines, chemokines, and adhesion molecules. Cerebral ischemic injury and blood flow reperfusion can cause inflammatory cascades, including oxidative stress, excitotoxicity, and inflammatory cell infiltration, as well as the production of cytokines and chemokines, which further lead to nerve tissue injury and apoptosis [165]. The regulation of NF-κB activity by Nrf2 is manifested in inhibiting inflammatory responses. Studies have shown that Nrf2 can regulate the TLR4/MyD88/NF-κB pathway in CIRI models and play a neuroprotective role [140]. Another target gene of Nrf2, p62, can regulate antioxidant and inflammatory activities. P62, as a protein scaffold, enhances the activity of Nrf2 by mediating the autophagic degradation of Keap1. P62 has oligomerization and can promote the ubiquitination and activation of TNF receptor-related factor 6 (TRAF6), enhance nerve growth factor NGF, and mediate the NF-κB signaling pathway [166].

The NOD-like receptor protein 3 (NLRP3) inflammasome is a protein complex located in the cells. Its main function is to activate the production of caspase-1, IL-1, and IL-18, thus promoting inflammation and apoptosis, and causing neuronal damage [167]. The NLRP3 inflammasome plays an important role in ischemia-induced inflammatory injury, and thioredoxin interacting protein (TXNIP) is closely related to the activation of NLRP3. Various factors such as ROS, toll-like receptor (TLR) agonists, and pro-inflammatory cytokines can promote NLRP3 expression [168]. It has been shown that knockdown of the NADPH oxidase subunit or free radical scavenger can inhibit ROS production and thus reduce NLRP3-induced IL-1 β, which reveals that ROS plays a key role in inflammasome activation [169]. Bruton’s tyrosine kinase (BTK) is a class of tyrosine kinases involved in the activation of the NLRP3 inflammatory complex in brain ischemia and reperfusion, resulting in the expression of mature caspase-1, as well as increased levels of IL-1. An inhibitor of BTK, ibrutinib, was found to inhibit the activation of the NLRP3 inflammatory complex and reduce IL-1 expression in the brain I/R model [170]. The study showed that Pleckstrin homology-like domain, family A, member 1 (PHLDA1) effectively alleviates CIRI by inhibiting the activation of NLRP3 [171]. Study has shown that Nrf2 downregulates NLRP3 inflammasome activity by acting on thioredoxin-1 (Trx1)/thioredoxin interacting protein (TXNIP) complex, thereby inhibiting inflammatory response and playing a protective role in CIRI [172]. Increasing evidence suggests an interaction between Nrf2 and the inflammasome. Administration of the Nrf2 activator tert-butyl hydroquinol (t BHQ) significantly reduced the expression of TXNIP and NLRP3 inflammasome, as well as the downstream factors caspase-1, IL-1 β,and IL-18 after t MCAO [172].

Evidence suggests that ischemic stroke triggers a range of cellular responses, including the accumulation of inflammatory cells in systemic circulation. The progress is linked to stroke-related secondary brain injury and can lead to further aggravation of infarction. It is currently known that the expression of Nrf2/HO-1 inhibits cellular inflammation and thus plays a neuroprotective role in the course of stroke [173]. Local inflammatory processes in the ischemic brain tissue can cause damage to the brain tissue. In addition, recent studies showed that peripheral inflammation also plays an important role in stroke injury. For instance, in rats suffering from MCAO, the spleen size was decreased after a stroke, mainly due to the upregulation of catecholamines causing the release of monocytes, and neutrophils [174]. Although most of the damage is caused by inflammation itself, the ensuing immunosuppression also poses non-negligible challenges. Immunosuppression is a response to inflammation after stroke, and it can trigger a variety of infections, such as pneumonia and urinary tract infections [175]. The role of Nrf2 in peripheral inflammation after stroke is still an exploratory topic, and further research is needed to understand its interaction and mechanism in the peripheral inflammatory organs of the body.

### 5.3. Nrf2 and Regulation of Mitochondrial Function

Mitochondria are the sites of oxidative metabolism in eukaryotes, and the sites where sugars, fats, and amino acids are finally oxidized to release energy. Study has shown that mitochondrial damage is one of the mechanisms of cell death [176]. Studies have shown that acute ischemia and hypoxia can cause mitochondrial dysfunction [177]. The physiological function of mitochondria is important for adult neurogenesis, which helps to repair neuronal cells damaged by cerebral ischemia. Neurogenesis requires sufficient ATP to provide energy. As the main source of cellular ATP, healthy mitochondrial function is an important prerequisite for effective neurogenesis [178]. The balance of mitochondrial fusion and division plays a crucial role in maintaining the normal function of neurons. CIRI can disrupt the balance of mitochondrial fusion and division by regulating the expression and modification of fusion- and division-related proteins, thereby disrupting the homeostasis of the intracellular environment and leading to neuronal death [179]. Many studies have shown that mitochondrial dysfunction is involved in the process of ischemic injury, so we can speculate that ischemic injury can be alleviated by reducing mitochondrial dysfunction. In the process of ischemic stroke, the lack of nutrition and oxygen promotes cell death [180]. After cell death, it promoted the production of ROS, which in turn aggravates mitochondrial dysfunction in feedback [181]. At the same time, mitochondrial dysfunction can lead to severe energy deficiency, increase ROS production in neurons, and ultimately lead to cell death. Most intracellular ROS are produced in mitochondria, mainly by complexes I and III of the mitochondrial electron transport chain. Mitochondria stress is accompanied by the accumulation of unfolded proteins, oxidative phosphorylation, fatty acid oxidation, and other pathological processes [182].

Activation of Nrf2 leads to mitochondrial biogenesis and the enhancement of mitochondrial antioxidant reaction [183,184]. Although the activation of Nrf2 is usually related to the induction of mitochondrial-localized antioxidant enzymes, its regulatory mechanism is not fully understood. Nrf2 is able to directly bind nuclear respiratory factor 1 (Nrf1) and the promoter region of PTEN-induced putative kinase 1 (PINK1) [185], which are mainly responsible for mitochondrial biogenesis and quality control, respectively. Some studies have shown that ROS induced the activation of Nrf2 in some way [186]. In one study, researchers found a novel biscoumarin compound, called COM3, which has substantial antioxidant effects in neurons. Study showed that COM 3 can improve neuronal mitochondrial energy metabolism after experiencing oxidative stress caused by oxygen-glucose deprivation (OGD) or MCAO. The mechanism may be that COM 3 activates nuclear transcription of Nrf2 by interfering with Keap1, thereby balancing endogenous redox activity and restoring mitochondrial function [187]. Notoginsenoside R1 (NGR1) is a novel phytoestrogen that is isolated from Panax notoginseng. The researchers found that NGR1 showed a protective effect on cerebral I/R injury in vivo and in vitro. The neuroprotective mechanism of NGR1 may be to inhibit mitochondrial dysfunction by regulating the ER-dependent Akt/Nrf2 pathway. In addition, Nrf2 activation can increase the expression of antiapoptotic protein Bcl-2 and inhibit the translocation of Bcl-2-related x (Bax) protein to mitochondria, thus alleviating the release of mitochondrial cytochrome c and the activation of downstream apoptotic proteases [188]. In conclusion, Nrf2 is a potent mediator to inhibiting mitochondrial dysfunction of cerebral ischemia through multiple mechanisms.

### 5.4. Nrf2 and Protection of Blood–Brain Barrier Function

The endothelial blood–brain barrier (BBB) represents a barrier between the circulation and the CNS compartment [189]. The normal structure and function of the BBB is the key to maintain the stability of the central nervous system. BBB injury occurs soon after cerebral ischemia [190]. Cerebral ischemia-reperfusion can damage the integrity of the blood–brain barrier and increase the permeability, further aggravating the damage of ischemic brain tissue [191]. The destruction of the BBB after cerebral ischemia leads to the infiltration of inflammatory factors and cells, which in turn leads to brain edema [192]. Ischemic preconditioning (IPC) can protect the blood–brain barrier, and the mechanisms underlying IPC-mediated protection of the BBB involve VEGF, ERK, or inflammatory pathways [193]. Tight junctions (TJs) and adherens junctions (AJs) play a critical role in maintaining BBB integrity. In a study, IPC directly upregulated the TJ protein claudin 5 and the AJ protein CDH5 [194]. The content of ROS increased after cerebral ischemic stroke; related research has shown that ROS production increased BBB permeability [195]. MMPs could destroy tight junctions of the BBB [196].

In in vivo experiments, the Nrf2 activator dimethyl fumarate (DMF) can prevent the destruction of tight junctions between endothelium and reduce the activity of matrix metalloproteinase in brain tissue. In in vitro experiments, DMF helps to maintain endothelial tight junctions, inhibit the expression of inflammatory cytokines, and weaken leukocyte migration. However, knockdown of Nrf2 expression aggravated the delocalization of tight junction protein ZO-1 under ischemia and weakened the protective effect of DMF, indicating the protective role of Nrf2 in BBB integrity [197]. Another study showed that in response to H/R stress, the expression of Abcc1, Abcc2, and Abcc4 mRNA in the BBB increased, and the expression of Abcc gene was regulated by the Nrf2 pathway [198].

Sulforaphane treatment before stroke activates the Nrf2 pathway. BBB disruption and brain damage were reduced by sulforaphane treatment. The authors believe that if the Nrf2 defense pathway in the brain microvascular system can be activated, it will help to prevent BBB disruption and neurological dysfunction in ischemic stroke [199]. Other Nrf2 activators, such as sulforaphane (SFN), have also shown protective effects on the blood–brain barrier [200]. Nomilin (NOM) is a limonoid compound obtained from the extracts of citrus fruits. NOM protects against cerebral I/R-induced neurological deficits and BBB disruption by regulating the Nrf2 pathway [201]. Astragaloside IV (ASIV) is isolated from Astragalus membranaceus. A study showed that ASIV protected the integrity of the BBB, and the Nrf2 pathway is involved in this process [202]. The destruction of the BBB after ischemic stroke permits peripheral inflammatory factors or bacteria to easily enter brain tissue, leading to brain injury. Therefore, focusing on the protection of the BBB through Nrf2 should be further studied.

### 5.5. Nrf2 Regulates Ferroptosis

Ferroptosis is a recently identified nonapoptotic-programmed cell death process. Ferroptosis is a form of cell death that depends on iron and oxidative stress [203]. It is characterized by increased lipid peroxidation, which leads to cell death by destroying the integrity of the cell membrane. The pathological process of ferroptosis can be regulated by glutathione peroxidase 4 (GPX4). Study showed that oxidative stress and iron metabolism are both closely related to ferroptosis initiation [204]. Further studies showed that ferroptosis can be inhibited by iron chelators, iron intake inhibitors, and lipophilic antioxidants [205]. Now, scholars believe that the main cause of cell death caused by ferroptosis is the inactivation of the cellular antioxidant system, the decrease of cellular antioxidant capacity, and the accumulation of intracellular lipid ROS leading to cell death [206].

Studies have shown that ferroptosis plays a key role in cerebral I/R injury. Moreover, inhibition of ferroptosis can play a neuroprotective role [207]. Furthermore, studies have shown that ferroptosis was detected in a mouse model of cerebral ischemia. The application of ferroptosis inhibitors significantly reduced brain injury [208]. Chen et al. [209] analyzed and identified ferroptosis-related differentially expressed genes (DEGs) in ischemic stroke by bioinformatics. A study showed that acute cerebral ischemia induces neuronal ferroptosis. Administration of drug therapy can alleviate cerebral ischemia injury, and its mechanism includes inhibition of ferroptosis through the transferrin receptor 1/divalent metal transporter 1(TFR1/DMT1) and solute carrier family 7 member 11/glutathione peroxidase 4(SCL7A11/GPX4) pathways [210].

A previous study showed that proper activation of Nrf2 can promote the reduction of cerebral ischemia injury [211]. Numerous enzymes and proteins involved in lipid peroxidation are Nrf2 target genes, including those involved in iron metabolism [212], such as FPN and heme-oxygenase 1 (HMOX-1) [213]. Furthermore, Nrf2 is strongly associated with the ferroptosis oxidative stress pathway. Studies have shown that ferroptosis is involved in the process of injury induced by ischemia-reperfusion. The protective effect of BCP on ischemic brain injury is related to the regulation of ferroptosis, and its mechanism is related to the activation of the Nrf2/HO-1 pathway [151]. Another study showed that rehmannioside A can improve cognitive impairment after ischemic stroke, which may be related to inhibition of neuroptosis and activation of the PI3K/AKT/Nrf2 pathway [112].

## 6. Drugs or Compounds Affect Cerebral Ischemic Stroke by Regulating Nrf2 Signaling Pathways

In recent years, more and more people have found that natural compounds extracted from plants can protect against cerebral ischemia. These compounds have anti-inflammatory, antioxidation, and antiapoptosis effects [214]. We speculate that it is possible to screen out drugs for treating or preventing cerebral ischemia injury from these compounds. There have been many studies on the role of Nrf2 in cerebral ischemic stroke. It has been found that regulating Nrf2 and related signaling pathways or mechanisms can alleviate cerebral ischemic stroke injury. In some studies (Table 2), cell survival was improved by targeting Nrf2-related signaling pathways.

## 7. Concluding Remarks

After cerebral ischemic stroke, the degree of functional damage of nerve cells depends on the degree of insufficient tissue perfusion. The purpose of cerebral ischemic stroke treatment is to preserve the neuronal function in ischemic penumbra as much as possible. Nrf2 plays an important role in maintaining the redox homeostasis of cells. In this review, we introduced the signaling pathway for regulating Nrf2 in the process of cerebral ischemic stroke. In addition, the moderate activation of Nrf2 is beneficial to reduce brain tissue damage after cerebral ischemia, involving mechanisms such as antioxidative stress, anti-inflammation, regulation of mitochondrial function, protection of the blood–brain barrier, regulation of iron death, etc., which can reduce neurological deficit. To summarize, we maintain that Nrf2 is a valuable therapeutic target.

In the future, we need to clarify the specific mechanism behind these phenomena through more in-depth research. For example, does the specific role of Nrf2 in ischemic stroke protect or exacerbate ischemic stroke? What is the role of Nrf2 in different cell types? What is the role and regulatory mechanism of Nrf2 in neuronal cells and microglia? The role and mechanism of Nrf2 in peripheral inflammation or immune organs deserve further study. Recently, there are many studies on the role of ferroptosis in ischemic stroke. What is the relationship between Nrf2 and ferroptosis in the process of ischemic stroke and reperfusion injury? What are the specific regulatory mechanisms? Further researches are needed. How does ferroptosis lead to cell death after ischemic stroke? Is it involved in the process of neuronal cell death? Further research is needed. In addition, what is the relationship between ferroptosis and oxidative stress? None of this is known yet. In addition, there are many studies on the mechanism of oxidative stress, but few studies on the interaction between oxidative stress and endoplasmic reticulum stress. We believe that the interaction between these processes deserves further study. Solving these problems will promote the treatment of ischemic stroke. More researches are needed to transform this therapy from laboratory to clinical.

More and more evidence shows that the activation of Nrf2 by some Chinese herbs and may be a promising therapeutic method for ischemic stroke. However, the specific activation pathway of Nrf2 and the specific mechanism of alleviating cerebral ischemia and its substrates still need to be further studied. In addition, the time window in which these drugs function requires more careful study. In conclusion, the treatment targeting Nrf2 in cerebral ischemic stroke is a promising field, and the development of drugs targeting Nrf2 has important clinical significance for the treatment of cerebral ischemic stroke.

## Figures and Tables

**Table 1:** Several traditional Chinese medicine/compounds affect cerebral ischemic stroke by regulating Nrf2 signaling pathways.

Medicine	Model (s)	Related Signaling Pathway	Related Mechanism	Effect	Reference
Dihydrocapsaicin (DHC)	MCAO/R	Activate SOD and GPx, downregulate ROS, NOX2, NOX4, NF-ĸB, NO, and MMP-9	Attenuate oxidative stress and inflammation	Protective	[73]
Acteoside (ACT)	MCAO/R	Decrease ROS and MDA, increase SOD and CAT	Attenuate oxidative stress and neuronal apoptosis	Protective	[74]
Schizandrin A (Sch A)	MCAO/R and OGD/R	Downregulate iNOS, COX-2, IL-1β, IL-6, and TNF-α, increase SOD, CAT, HO-1 and NQO-1	Suppress inflammation and oxidative stress	Protective	[75]
Icariside II	MCAO/R	Decrease ROS and MDA, increase SOD, GSH-Px, catalase, Nrf2, and HO-1	Attenuate oxidative stress	Protective	[76]
Rutaecarpine (Rut)	MCAO/R	Alleviate IL-6, IL-1β, LDH, MDA, and ROS, increase IL-4, IL-10, SOD, HO-1, and NQO1	Alleviate inflammatory response and oxidative stress	Protective	[77]
Biochanin A	MCAO/R	Activate SOD, GSH-Px, and HO-1, suppress MDA, NF-kB	Antioxidative and anti-inflammatory actions	Protective	[78]
Theaflavin	MCAO/R and OGD/R	Decrease ROS and MDA, increase SOD and GSH-Px	Restore the impaired antioxidant defense system	Protective	[79]
Carvacryl acetate (CA)	MCAO/R	Decrease ROS and MDA, increase SOD	Antioxidant stress	Protective	[80]
Convolvulus pluricaulis Choisy	BCCA	Increase SOD, catalase, glutathione, and total thiol	Protect against oxidative damage	Protective	[81]
Cepharanthine (CEP)	MCAO/R and OGD/R	Decrease ROS and MDA, NLRP3, ASC, and cleaved caspase-1, increase SOD	Inhibit microglia activation, inflammation, and reduce oxidative stress	Protective	[82]
Sanggenon C (SC)	MCAO/R and OGD/R	Decrease TNF-α, IL-1β, IL-6, ROS, and MDA, increase SOD	Inhibit inflammation and oxidative stress	Protective	[83]
Coicis Semen	MCAO/R and OGD/R	Decrease ROS and MDA, increase SOD, GSH-Px, ZO-1 and occludin, CD31, and VEGF	Inhibit oxidative stress and promote angiogenesis	Protective	[84]
Piceatannol (Pic)	MCAO/R	Decrease ROS, increase SOD, CAT, and GSH-Px	Suppress oxidative stress	Protective	[85]
Garcinol	MCAO/R and OGD/R	Inhibit IL-1β, IL-6, TNF-α, NF-κB, MDA, and nitric oxide (NO), increase SOD	Attenuate inflammation and oxidative stress	Protective	[86]
Fisetin	MCAO/R and OGD/R	Decrease IL-1, TNF-α, iNOS, IL-1β, COX-2, IL-6, and PGE2	Inhibit inflammation and oxidative stress	Protective	[87]
Geraniin	MCAO/R and OGD/R	Decrease LDH, NO, nNOS and MDA, increase SOD	Decrease oxidative stress and neuronal apoptosis	Protective	[88]
Cucurbitacin B (CuB)	MCAO/R and OGD/R	Decrease LDH, ROS, and NLRP3	Inhibit oxidative stress and inflammation	Protective	[89]
Fraxin	MCAO/R and OGD/R	Decrease ROS, NF-κB, IKK-β, p38 MAPK, ERK1/2, and Keap1	Inhibit oxidative stress, inflammatory response, and cell apoptosis	Protective	[90]
Scutellarin	tMCAO	Decrease ROS, 4-HNE, 8-OHDG, NT-3, PARP1, NOX1, NOX2, and NOX4	Suppress oxidative stress	Protective	[91]

**Table 2:** Several traditional Chinese medicine/compounds affect cerebral ischemic stroke by regulating Nrf2 signaling pathways.

Medicine	Model (s)	Related Signaling Pathway	Related Mechanism	Effect	Reference
β-Caryophyllene (BCP)	MCAO/R and OGD/R	Activate Nrf2/HO-1 pathway	Protect against ferroptosis	Protective	[151]
Diosmetin	MCAO/R and OGD/R	Activate Keap1/Nrf2/ARE pathway	Attenuate oxidative stress and inflammation	Protective	[215]
Dl-3-n-butylphthalide (NBP)	Repeated CIRI	Nrf2-modulated TLR4/MyD88/NF-κB pathway	Antioxidant, antineuroinflammatory	Protective	[140]
Edaravone dexborneol	Repeated CIRI	Activate Nrf2/HO-1 pathway	Antioxidant, antineuroinflammatory	Protective	[216]
Rhodiola sacra	tGCI	Activate AMPK/Nrf2 pathway	prevent oxidant stress	Protective	[217]
Geraniin	MCAO/R and OGD/R	Activate Nrf2/HO-1 pathway	Suppress oxidative stress and neuronal apoptosis.	Protective	[88]
Cajaninstilbene acid (CSA)	MCAO/R and OGD/R	Activate AMPK/Nrf2 pathway	Reduce oxidative stress and mitochondrial disfunction	Protective	[218]
Thymus quinquecostatus Celak	tMCAO	Activate Keap1/Nrf2/HO-1 pathway	Antioxidant stress	Protective	[105]
Isorhapontigenin (ISO)	MCAO/R and OGD/R	Activate PKCε/Nrf2/HO-1 pathway	Protect against oxidative damage	Protective	[219]
Palmatine (PAL)	tMCAO	Activate AMPK/Nrf2 pathway	Reduce oxidative stress and inflammatory response	Protective	[220]
Pelargonidin	MCAO/R	Activate Nrf2/HO-1 pathway	Reduce oxidative stress and inflammatory response	Protective	[147]
Eriocitrin	MCAO/R	Activate Nrf2/HO-1/NQO1/NF-κB pathway	Attenuate oxidative injury and inflammatory response	Protective	[139]
Lupeol	MCAO/R	Involve Nrf2 and P38 MAPK modulation	Suppress oxidative stress and inflammatory response	Protective	[129]
A biscoumarin compound COM 3	MCAO/R and OGD/R	Modulate Nrf2/Keap1/ARE pathway	Antioxidant stress	Protective	[187]
Chlorogenic acid (CGA)	CI/R model	Activate Nrf2/HO-1 pathway	Regulate oxidative stress	Protective	[221]
Lyciumamide A (LyA)	MCAO/R and OGD/R	Activate PKCε/Nrf2/HO-1 pathway	Ameliorate oxidative damage and neuronal apoptosis	Protective	[222]
Nomilin	MCAO/R and OGD/R	Activate Nrf2/NQO1 pathway	Mitigate oxidative stress	Protective	[201]
Rutaecarpine (Rut)	MCAO/R	Activate Nrf2/HO-1 pathway	Inhibit apoptosis, inflammation, and oxidative stress	Protective	[77]
Swertiamarin (Swe)	MCAO/R and OGD/R	Activate Nrf2/HO-1 pathway	Suppress oxidative stress	Protective	[223]
Ginkgolides and bilobalide	MCAO/R and OGD/R	Mediate Akt/Nrf2 pathway	Inhibit oxidative stress	Protective	[109]
Icariside II	MCAO/R	Activate Nrf2/HO-1 pathway	Inhibit oxidative stress	Protective	[76]
Schizandrin A (Sch A)	MCAO/R and OGD/R	Regulate AMPK/Nrf2 pathway	Suppress inflammation and oxidative stress	Protective	[75]
Luteoloside	MCAO/R	Regulate PPARγ/Nrf2/NF-κB pathway	Inhibit neuroinflammation	Protective	[138]
Total Flavonoids from A. esculentus	TCI-RI model	Activate Nrf2-ARE pathway	Inhibit oxidative stress	Protective	[224]
Diterpene ginkgolides	MCAO/R and OGD/R	Activate PI3K/Akt/Nrf2 pathway	Inhibit oxidative stress	Protective	[113]
Artesunate	CI/RI model	Activate MAPK/Nrf2 pathway	Suppress oxidative stress and inflammatory process	Protective	[128]
6’-O-galloylpaeoniflorin (GPF)	MCAO/R and OGD/R	Activate PI3K/Akt/Nrf2 pathway	Prevent oxidative stress, inflammation, and apoptosis	Protective	[114]
Liraglutide	MCAO/R	Activate Nrf2/HO-1 pathway	Antioxidant stress	Protective	[225]

tGCI: transient global cerebral ischemia; PKC-ε: protein kinase C ε; TCI-RI: transient cerebral ischemia-reperfusion injury.
